# Baicalin: Natural Sources, Extraction Techniques, and Therapeutic Applications Against Bacterial Infections

**DOI:** 10.3390/molecules30173464

**Published:** 2025-08-22

**Authors:** Xin Meng, Chao Ning, Mengna Kang, Xiuwen Wang, Zhiyun Yu, Xueyu Hao, Haiyong Guo

**Affiliations:** 1 College of Life Science, Jilin Normal University, Siping 136000, China; 18624395651@163.com (X.M.); 18643477064@163.com (C.N.); kang000719@163.com (M.K.); liaoshiwxw@163.com (X.W.); yuzhiyun0808@163.com (Z.Y.); 2 College of Food and Biology, Changchun Polytechnic, Changchun 130012, China; haoxueyu1990@163.com

**Keywords:** baicalin, enzyme-assisted extraction, gastrointestinal infections, drug combination therapy

## Abstract

The emergence of bacterial strains resistant to available antibiotics due to overprescription has prompted a search for alternative treatments. Among the most promising is baicalin, a flavonoid extracted from the roots of *Scutellaria baicalensis*. Roots, the primary natural source of baicalin, have been extensively explored using emerging extraction technologies such as ultrasonic-assisted extraction and supercritical fluid extraction. These methods offer significant advantages over traditional reflux extraction for baicalin preparation, including shorter extraction times, lower energy consumption, and improved environmental sustainability. Baicalin exhibits remarkable antibacterial activity in vitro and has demonstrated therapeutic efficacy against gastrointestinal infections, meningitis, pulmonary diseases, and sepsis, among other infectious disorders, in animal models. Documented mechanisms of action include disrupting the *Escherichia coli* membrane, downregulating quorum-sensing gene expression in *Pseudomonas aeruginosa*, and inhibiting host inflammatory pathways such as PI3K/Akt/NF-κB. However, its clinical translation faces several bottlenecks, including reliance on animal experiment data, low bioavailability, and regulatory compliance issues. This review compares baicalin extraction yields from different natural sources, summarizes the advantages and disadvantages of various extraction technologies, analyzes possible mechanisms of action in treating different bacterial diseases, and discusses outstanding challenges and best strategies for expanded clinical use against bacterial infection. Our aim is to provide a valuable reference for future research and clinical applications.

## 1. Introduction

Bacterial infection remains a leading cause of death globally, and antimicrobial resistance (AMR) has further complicated treatment. It is estimated that AMR contributed to 4.95 million deaths worldwide in 2019, with 1.27 million of these deaths directly attributable to the inefficacy of available antibiotics [1]. The rise in AMR, particularly the emergence of bacterial strains resistant to multiple antibiotics, has prompted the search for nontraditional alternative antimicrobial agents. Among the most promising are plant extracts, particularly flavonoid compounds and their derivatives such as isocoumarins and glycosides [2]. These compounds have become key targets for antimicrobial drug development due to demonstrated bactericidal activity and their complementary biological activities against infection, such as anti-inflammatory and anticancer effects. Furthermore, plant flavonoids possess unique pharmacological regulatory potential conferred by phenolic hydroxyl groups and glycosidic bonds [3], suggesting that their bioactivities can be further enhanced through rational chemical modifications.

**Figure 1:**
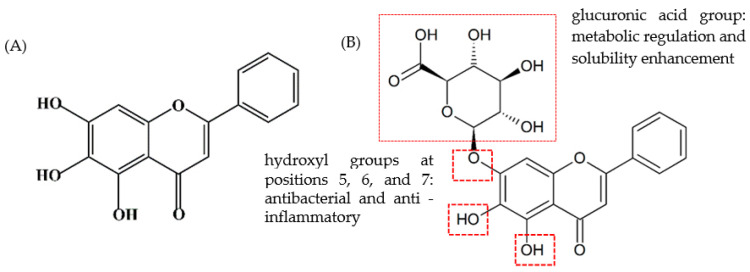
The chemical structures of baicalein (**A**) and baicalin (**B**).

Among the best-studied and most promising plant flavonoids for therapeutic applications is baicalin (chemical formula: C_21_H_18_O_11_), which is derived primarily from the dried roots of *Scutellaria baicalensis* (Chinese skullcap) [4], a perennial flowering plant of the *Lamiaceae* family native to wide regions of Southeast Asia. Trace amounts of baicalin have also been detected in the roots of related species of the same genus, such as *Scutellaria integrifolia* and *Scutellaria racemosa*, but *S. baicalensis* remains the core natural source for industrial extraction [4]. Baicalin is isolated as a yellow crystalline solid with a molecular weight of 446.4 g/mol and a melting point of 202–205 °C [5]. It is almost insoluble in water but readily soluble in alkaline solutions, with pKa_1_ and pKa_2_ values of 7.6 and 10.1 in 0.1 M NaCl at 25 °C [5]. The core structure of baicalin is baicalein (chemical formula:C_15_H_10_O_5_), a phenol-containing aglycone with a 7-position hydroxyl group linked to glucuronic acid via a glycosidic bond, which enhances its solubility in polar protic solvents (Figure 1) [6]. After administration, free baicalin accumulates at relatively high levels in the kidneys, while relatively low concentrations are initially detected in the heart, liver, and spleen [7]. In contrast, liposome-encapsulated baicalin concentrations progressively increase in the heart, liver, spleen, lungs, and brain, concomitant with decreasing concentrations of free baicalin in the kidneys [8]. Following oral ingestion, baicalin is hydrolyzed by the intestinal flora to form baicalein, which is then absorbed. Subsequently, baicalein is converted back to baicalin in the liver, resulting in biphasic absorption peaks [7,8]. After intravenous administration, baicalin exhibits a typical pharmacokinetic curve; however, the free form is cleared rapidly, with a short half-life [7]. Both the parent drug and its conjugated forms (such as glucuronides and sulfates) can be detected in the serum [9], while baicalin metabolism is dominated by glucuronidation. Due to first-pass metabolism, glucuronic acid conjugates predominate in plasma [9]. The ileum and jejunum also participate in the conjugate metabolism of baicalin [9]. The glucuronide and sulfate conjugates generated through metabolism are primarily excreted into the small intestine via the biliary route, with renal excretion serving as a secondary pathway [9].

Baicalin is insoluble in alcohols but soluble in organic solvents such as chloroform and dimethyl sulfoxide (DMSO); therefore, the latter are most often used for traditional extraction [10]. However, traditional extraction methods such as thermal reflux have limited efficiency because the prolonged high temperatures required may induce degradation of the target compound. In addition, reflux extraction requires continuous heating of the solvent to maintain evaporation–condensation cycles, a process that consumes substantial energy and generates significant greenhouse gas emissions, contributing to global warming [11]. In recent years, new technologies have been developed to improve extraction efficiency and reduce environmental impacts. These included ultrasonic-assisted extraction [12], deep eutectic solvent-based ultrahigh-pressure extraction [13], microwave-assisted extraction [14], and enzyme-assisted extraction [15]. For example, the yield obtained by deep eutectic solvent–ultrahigh-pressure extraction (116.8 mg/g) was 5.8% higher than that of reflux extraction using 70% ethanol, while total extraction time was reduced by 97.8% [13]. Furthermore, deep eutectic solvents are biodegradable and have low toxicity, enhancing the overall extraction efficiency and environmental sustainability [16].

The antibacterial efficacy of baicalin is conferred mainly by a carboxyl group formed through conjugation of the C7 hydroxyl with glucuronic acid, which disrupts membrane structural integrity and regulatory functions [16]. The electron-rich carboxyl group interacts with positively charged groups on the bacterial phospholipid bilayer or membrane proteins, inserting into the membrane and disrupting its integrity. For example, baicalin induces leakage of intracellular substances such as potassium ions, DNA, alkaline phosphatase, and β-galactosidase from *E. coli* [17]. In addition, the carboxyl group interferes with the membrane-associated quorum-sensing (QS) system by interacting with membrane receptors or signaling molecules, thereby hindering QS signal transduction. In *Pseudomonas aeruginosa*, baicalin downregulates QS regulatory gene expression and reduces the production of QS signaling molecules, which in turn inhibits biofilm formation and the release of virulence factors [18]. Based on these mechanisms, baicalin has demonstrated therapeutic potential against bacterial diseases such as gastrointestinal infections [19], meningitis [20], pulmonary diseases [21], and sepsis [22] in both in vitro and animal models. Furthermore, baicalin efficacy against bacterial infections can be enhanced when used in combination with conventional antibiotics such as tobramycin [16]. However, multiple challenges remain for its routine clinical application, including limitations imposed by its physicochemical properties and bioavailability. For instance, poor water solubility limits baicalin extraction efficiency. Moreover, limited membrane permeability results in extremely low absolute bioavailability, particularly following oral administration (2.2%) [6,23]. The bioavailability of baicalin can be enhanced through approaches such as nanoformulation preparation [7], prodrug design [24], and complexation with carriers [25], suggesting feasible strategies for oral therapeutic use. For example, the bioavailability of a baicalin–phospholipid complex in a self-microemulsifying drug delivery system reached 220.37% of that of free, unmodified baicalin [6]. This review focuses on the following topics: (1) the best natural sources of baicalin, (2) improved extraction techniques, (3) the potential therapeutic value of baicalin preparations, and (4) current challenges to clinical use. This information is presented to facilitate further basic research, formulation development, and clinical application trials.

## 2. Natural Sources of Baicalin

Baicalin is a major plant 4′-deoxyflavone that varies substantially in content across species and even among different parts of the same plant (Table 1). In *S. baicalensis*, baicalin is present primarily in the roots. Costine et al. reported a fresh weight of 26.05 ± 3.9 mg/g of root tissue [4], while Xu et al. reported a dry weight content in roots as high as 84.21 mg/g, 22 times higher than in flowers, 100 times higher than in stems, and 56 times higher than in leaves [26]. The root is also the main site of baicalin biosynthesis. Silencing of the key biosynthetic enzyme gene *SbFNSII-2* markedly reduced root baicalin content [27]. In contrast, Costine et al. reported that baicalin was present mainly in the leaves of *S. lateriflora*, with a fresh weight content of 11.66 ± 2.9 mg/g [4]. Tuan et al. similarly found that the baicalin content was highest in the leaves of *S. lateriflora* (dry weight 33.58 mg/g), followed by the roots (14.91 mg/g), petioles (5.71 mg/g), and stems (4.7 mg/g) [28]. Alternatively, baicalin content was found to be extremely low in the roots, stems, and leaves of *S. arenicola* (0.03, 0.01, and 0.04 mg/g dry weight, respectively) and *S. integrifolia* (0.02, 0.01, and 0.01 mg/g, respectively) [4]. Baicalin is also present in other plant genera. The total baicalin content in different tissue samples of *Siegesbeckia pubescens* Makino varied greatly, ranging from 0.14 mg/g in one Hebei sample, to 0.099 mg/g in a Shenyang sample, 0.082 mg/g in an Anhui sample, and 0.006 mg/g in a second Hebei sample [29]. The above-ground parts of the three *Veronica* species (*Plantaginaceae*) also contain baicalin, with the highest content in *V. urticifolia* (0.779 mg/g dry weight), followed by *V. teucrium* (0.347 mg/g dry weight) and *V. jacquinii* (0.009 mg/g dry weight) [30]. Baicalin was also detected in the seeds, young fruits, and flowers of *Oroxylum indicum*, with measured contents of 68.7 ± 1.1 mg/g in seed extract, 68.2 ± 0.4 mg/g in orange-red crystals isolated from the plant, 16.2 ± 0.4 mg/g in a yellow precipitate, 1.9 mg/g in young fruit extract, and 0.4 mg/g in flower extract [31]. In addition, baicalin has been detected in the roots, stems, and leaves of *S. wrightii*, *S. tomentosa*, and *S. racemose* [32]. For instance, the dry weight content was highest in the roots of *S. wrightii* at 122.14 ± 1.42 mg/g (~5 times that of the commonly used *S. baicalensis*), the dry weight content in *S. tomentosa* roots was 17.30 ± 0.22 mg/g (with 10.63 ± 0.26 mg/g in the stems and 1.05 ± 0.03 mg/g in the leaves), and 11.11 ± 0.24 mg/g in the roots, 10.59 ± 0.11 mg/g in stems, and 15.21 ± 0.11 mg/g in the leaves of *S. racemosa* roots [32].

Notably, the baicalin content in *S. baicalensis* roots can also vary by geographic sampling location (Table 1). Cui et al. found that roots from an artificial cultivation site in Wangdu County, China (WDC site) contained ~180 mg/g dry weight baicalin, substantially higher than samples from several other sites; furthermore, contents differed markedly among wild samples from Chongli County, Zhangjiakou City, China (CLY, ≈160 mg/g), cultivated samples from Longhua County, Chengde City, China (LHZ, ≈150 mg/g), wild samples from LHY (≈140 mg/g), and wild samples from Chengde County, Chengde City, China (CDY, ≈100 mg/g) [33]. The baicalin contents in the leaves and stems were relatively low and varied with the sampling site. For instance, in CLY and CDY, baicalin content was significantly higher in the leaves than in the stems, whereas in LHY and WDZ, content was higher in the stem than the leaves. In LHZ, however, there was little difference in the content between the leaves and stem [33].

Baicalin content is also influenced by the pre-extraction treatment conditions. For instance, content was significantly higher in hairy root cultures of *S. lateriflora* treated with methyl jasmonate (MeJA) (up to 22.54 mg/g) than in untreated control cultures and significantly higher in hairy roots grown in the dark compared to those grown in the light [33]. Similarly, MeJA treatment doubled baicalin content in the roots of *S. baicalensis* compared with untreated controls [27]. Xu et al. reported that treatment with 200 μM MeJA for 72 h increased baicalin content in *S. baicalensis* suspension cells ~4.5-fold compared with controls (12.03 vs. 2.7 mg/g) [26]. Drought stress was also reported to enhance baicalin content by 68.72% in *S. baicalensis* samples treated with 10% polyethylene glycol (PEG) 6000 and by 35.86% in samples treated with 15% PEG 6000 [26]. Different LED light conditions and durations also impacted baicalin content in *S. baicalensis* seedlings [34]. Root content was highest at 100.42 ± 0.32 mg/g dry weight after 2 weeks of white light but was reduced after 4 weeks, while stem content was low overall, peaking at 0.17 ± 0.05 mg/g after 4 weeks of red light. Leaf content was also low but increased after 4 weeks of red light [34].

In conclusion, baicalin content varies by plant species, tissue type, and growth location/conditions and is further influenced by various experimental treatments. This diversity reflects the specificity of metabolic regulation formed during the evolution of different plants, which is influenced by multiple factors. Essentially, it is the result of the differentiation of metabolic regulatory networks driven by ecological adaptability. These findings indicate that there are abundant baicalin resources and diverse strategies for enhancing production, either by selecting specific high baicalin plant sources or by enhancing the baicalin content through optimized cultivation and treatment methods, these findings have important research value and may enhance the prospects of future clinical application.

## 3. Advances in Baicalin Extraction Technology

In the field of natural product research, efficient extraction is a major focus of natural product research. Various extraction techniques based on distinct principles have been developed and optimized for improved extraction efficiency, purity retention, and process applicability. In this section, we summarize the performance, advantages, and limitations of reflux extraction, water extraction, ultrasonic-assisted extraction, ultrahigh-pressure extraction, microwave-assisted extraction, and enzyme-assisted extraction (Table 2) to provide references for optimizing baicalin resources.

### 3.1. Reflux Extraction

Reflux extraction employs volatile organic solvents, such as ethanol, to create a continuous “evaporation–condensation” cycle [35]. In this process, the solvent containing plant material is heated in an extraction vessel, and the volatile solvent vapor containing the target active compound is then condensed and reintroduced into the extraction vessel until the active compound is fully extracted [35]. Li et al. obtained 155.5 ± 3.90 mg/g baicalin from *S. baicalensis* roots in 180 min using 50% aqueous ethanol heated to 90 °C [36]. However, this extraction time is significantly longer compared with ultrasonic-assisted extraction (23 min) or microwave-assisted extraction (10 min) [12,13]. In addition, reflux extraction equipment is typically large, and the process is not amenable to extensive automation, requires manual solvent addition, and involves a cumbersome purification process. In the chemical and pharmaceutical industries, the reflux method is suitable for raw materials that tolerate organic solvents and require higher temperatures and longer extraction times. For example, when extracting high-boiling-point spice components from plants, refluxing with a solvent such as ethanol can ensure a good yield [37].

### 3.2. Water Extraction

Water extraction uses water as the solvent to extract soluble components from solid or semisolid materials through heating or other means [38]. This method avoids the use of organic solvents and thus is more environmentally friendly [38]. However, it requires a large volume of water, which increases the processing costs, and the extract may contain impurities that necessitate further purification [38]. Baicalin has relatively low water solubility (67.03 ± 1.60 μg/mL) [6], a characteristic that makes it difficult to be fully dissolved during water extraction, thereby resulting in a low extraction rate. Li et al. determined the optimal parameters for extracting baicalin using water under reflux (although via single-factor experiments rather than an orthogonal design) and achieved a yield of 15.6 mg/g using a liquid–solid ratio of 25:1 mL/g, an extraction temperature of 93 °C, an extraction time of 2.4 h, and two extraction cycles [39]. In another study, Ni et al. optimized the conditions for extracting baicalin with water via orthogonal experiments (solid–liquid ratio 1:12, extraction time 30 min, soaking time 1 h) [40]. Under these conditions, the baicalin yield reached 32.7 mg/g, which was twice that obtained by Li et al. [39,40]. In the food and pharmaceutical industries, if high extract purity is not required and environmental protection is a priority, the water extraction method may be suitable. For example, in the initial extraction of traditional Chinese medicinal materials (such as honeysuckle for herbal teas), water extraction can produce large amounts of extract without introducing organic solvent pollutants into the environment [41].

### 3.3. Ultrasonic-Assisted Extraction

Ultrasonic-assisted extraction (UAE) leverages the mechanical, thermal, and cavitational effects of ultrasound to accelerate the release, diffusion, and dissolution of intracellular active compounds in tissue samples, thereby enhancing the extraction efficiency [42]. Under ultrasonic irradiation, cavitation bubbles rapidly form and collapse in the solvent, releasing shock waves that improve solvent penetration and mixing. The mechanical effects of ultrasound also allow the solvent to penetrate deeper into the sample matrix, thereby increasing the solid–liquid contact area and facilitating the release of intracellular products [43]. Liu et al. used UAE to extract baicalin from dried *S. baicalensis* powder. Under optimized conditions, including an 80-mesh particle size, 20:1 liquid-to-solid ratio, 57% ethanol as the solvent, 68 °C, a cycle duration of 66 min, and two extraction cycles, baicalin yield from *S. baicalensis* roots reached 12.95%. Relative to the baicalin content in untreated *S. baicalensis* roots (14.08%), this corresponds to an extraction efficiency of 92.0% [44]. UAE is simple, safe, rapid, efficient, energy-saving, and environmentally friendly if nontoxic solvents are used; however, it is unsuitable for large-scale production due to reduced ultrasonic propagation in large volumes [44]. Furthermore, not all natural products are amenable to ultrasonic extraction because some compounds may degrade. Zhang et al. found that gallic acid can be degraded during ultrasonic treatment, and similarly, catechins in tea can be degraded under certain ultrasonic conditions [45]. Moreover, the degradation rate of catechins increased with higher frequency and input power [46], likely because higher ultrasonic power generates free hydroxyl radicals that react with phenolic compounds, leading to their decomposition [47]. Therefore, when applying ultrasound, care must be taken to avoid excessive power that causes polyphenol degradation. Alternatively, UAE holds significant advantages for heat-sensitive components as ultrasound waves can quickly break down cell walls at relatively low temperatures [48]. For example, UAE has been effectively used to extract biological enzymes and peptides without significant activity loss. In the cosmetics industry, UAE can also improve the extraction efficiency of active components such as antioxidants and whitening agents from plants, thereby enhancing product quality [49].

### 3.4. Ultrahigh-Pressure Extraction

As the name implies, ultrahigh-pressure extraction (UHPE) uses extremely high pressure to disrupt plant cell walls and release active components into a solvent [50]. The high pressure forces solvent into the solid material, dissolving components that then diffuse out when the pressure is released [50]. As heating is not necessary, UHPE is energy efficient and achieves high yields by obviating the degradation of heat-labile components. This UHPE technique has been used to extract various phenolic and flavonoid compounds from plant tissue with high efficiency [13,51] and can also be used with deep eutectic solvents (DESs) containing a mixture of hydrogen bond acceptors and donors, offering benefits such as biodegradability and low toxicity [52]. Combining UHPE with DESs can further enhance the extraction efficiency. For instance, Wang et al. reported a baicalin yield of 116.8 mg/g using a DES composed of choline chloride and lactic acid (1:1 molar ratio) with 40% water content, a pressure of 400 MPa for 4 min, and a liquid–solid ratio of 110 mL/g, whereas a hot reflux aqueous extraction under similar conditions (80 °C, 133 mL/g, 3 h, 40% DES moisture) yielded only 84.3 mg/g [13]. This approach holds significant advantages for industries that require extracts with high bioactivity and quality, such as high-end health products and cosmetics. For components easily damaged by high temperature (e.g., baicalin and ginsenoside), UHPE enables efficient extraction in a low-temperature environment, maximizing the retention of biological activity [53].

### 3.5. Microwave-Assisted Extraction

Microwave-assisted extraction (MAE) involves the rapid heating of solvents and plant materials with microwave radiation, which facilitates the efficient release of target compounds from plant cells [54]. Microwave energy interacts directly with polar molecules, generating localized high temperatures and pressures and thereby enhancing the extraction efficiency [54]. This technique has been used to rapidly extract various active compounds, including essential oils [55], flavonoids [56], phenols [57], alkaloids [58], and glycosides [59], with reduced solvent usage and greater amenability to automation than traditional methods [60]. However, the extraction efficacy is strongly dependent on the microwave power, temperature, extraction duration, solvent type, and plant material properties [54]; therefore, optimization trials may be necessary for specific applications. The use of DESs can further enhance MAE efficiency due to their excellent microwave absorption properties [61]. One study reported that MEA using a hydrophobic DES (molar ratio 1:2 decanoic acid and N4444-Cl) yielded 106.96 mg/g baicalin from *S. baicalensis* (with 33% moisture content) at 85 °C and a 110 mL/g liquid–solid ratio, comparable to conventional methods (104.94 mg/g), while significantly reducing the extraction time from 3 h to just 10 min [60]. This DES–MAE combination is highly efficient and environmentally friendly although its applicability may be limited to heat-sensitive compounds or large-scale extraction. In practice, MAE is suitable for industrial production that requires rapid extraction and strict control of solvent usage, such as the extraction of food additives and natural pigments. It accomplishes extraction in less time and with less solvent when targeting compounds such as flavonoids and phenols [62].

### 3.6. Enzyme-Assisted Extraction

Enzyme-assisted extraction (EAE) is a novel method for isolating bioactive compounds that employs enzymes such as cellulase, pectinase, and hemicellulase to decompose the plant cell walls [63]. For example, endophytic microbial strains producing highly active cellulases can efficiently degrade plant cellulose, improving the extraction yield compared with commercial enzymes [64]. In one study on baicalin extraction assisted by cellulase from the endophytic strain HG-5, researchers optimized the conditions using carboxymethyl cellulose and sucrose as carbon sources and peptone and yeast extract as nitrogen sources to promote HG-5 growth and enzyme production. In addition, *E. coli* and *Saccharomyces cerevisiae* were added at the final stage of the enzymatic extraction to eliminate feedback inhibition by metabolites. The result was a baicalin yield of 1.56 g, 79.31% higher than that obtained from ethanol reflux extraction (0.87 g) [15]. Despite advantages such as shorter extraction times, high recovery, low solvent usage, and low energy consumption, EAE faces challenges for large-scale application, particularly the high cost of enzymes, incomplete hydrolysis, low enzyme activity under some conditions, and enzyme instability under others (such as higher temperatures) [65]. Modifications such as enzyme immobilization, recycling, or engineered robustness could significantly increase yields and improve product quality by permitting milder processing conditions. This EAE method is particularly applicable to bioactive compound extraction from plant materials with complex cell wall structures (such as the roots of *S. baicalensis* and other traditional medicinal herbs).

**Table 2:** Comparison of different technologies for baicalin extraction.

Extraction Technology	Extraction Conditions	Extraction Content	Advantages	Disadvantages	References
Reflux extraction	50% ethanol aqueous solution as the solvent, extraction at 90 °C for 180 min	155.5 ± 3.90 mg/g	high extraction efficiency; simple operation	long extraction time; high energy consumption; high temperatures may reduce pharmacological activity; environmental damage related to high energy consumption and toxic solvent use	[35,36]
Water extraction	solid–liquid ratio 1:12, extraction time 30 min, soaking time 1 h	32.7 mg/g	low raw material cost; simple operation; environmentally friendly	narrow application range (not suitable for fat-soluble substances); low purity yields	[38,40,41]
Ultrasound-assist-ed extraction (UAE)	80-mesh particle size, liquid–solid ratio 20:1, ethanol concentration 57%, temperature 68 °C, time 66 min, extracted twice	129.5 mg/g	high extraction rate, yield, and purity; environmentally friendly	high energy consumption; high temperatures may reduce pharmacological activity; extensive equipment requirements and high cost; need to optimize the extraction conditions	[43,44,45,46,47,48,49]
Ultra-high-pressure extraction (UHPE)	DES with a choline chloride to lactic acid molar ratio of 1:1, water content 40%, pressure 400 MPa, time 4 min, and liquid–solid ratio 110 mL/g	116.8 mg/g	high extraction efficiency; high purity; suitable for thermolabile compounds; environmentally friendly; low energy consumption	high cost; complex operation; restricted solubility; need for co-solvents; limited extraction efficiency	[13,52,53]
Microwave-assisted extraction (MAE)	hydrophobic DES with a DecA to N4444-Cl molar ratio of 1:2, water content 33%, temperature 85 °C, liquid–solid ratio 110 mL/g, extraction time 10 min	106.96 mg/g	high efficiency; solvent-saving; low energy consumption; strong selectivity; simple operation	high cost; operating conditions must be optimized; safety concerns	[54,60,61,62]
Enzyme-assisted extraction (EAE)	cellulase from the endogenous strain HG-5 (cultured with CMC/sucrose as the carbon source and peptone/yeast extract as the nitrogen source) for auxiliary extraction; *E. coli* and *S. cerevisiae* were added at the extraction end to eliminate metabolite feedback inhibition	1.56 g	mild extraction conditions; high extraction efficiency; high selectivity; environmentally friendly	high cost; enzyme instability and limited reuse; complex operating requirements	[15,65]

## 4. Therapeutic Efficacy of Baicalin Against Bacterial Diseases

Baicalin holds great potential for the treatment of infectious diseases owing to its multiple mechanisms of action, including inhibition of pathogen proliferation, modulation of inflammatory responses, and protection of tissue and organ function. Below, we detail the specific applications and action mechanisms of baicalin for gastrointestinal infections, bacterial meningitis, pulmonary diseases, and sepsis (Table 3), providing references for further clinical application and research on baicalin.

### 4.1. Applications of Baicalin for Gastrointestinal Infections

#### 4.1.1. Treatment of Enterotoxigenic *Escherichia coli* Infections

Enterotoxigenic *Escherichia coli* (ETEC) is a major cause of diarrhea. In 2016, ETEC was the eighth leading contributor to global diarrhea-related deaths, responsible for over 51,000 lives lost and accounting for ~3.2% of all diarrhea-related fatalities [66]. Pathogens inducing diarrhea produce two primary toxins, heat-stable enterotoxin (ST) and heat-labile enterotoxin (LT), which act by increasing cellular cGMP and cAMP levels, respectively, in host intestinal epithelial cells, leading to disruptions in gut ion and water transport [67]. The heat-stable toxin has also been found to reduce expression of the tight-junction proteins ZO-1 and claudin-1 by murine and porcine intestinal cells, thereby weakening barrier integrity. Moreover, ST shares structural homology with the paracrine hormones guanylin (GUCA2A) and uroguanylin (GUCA2B), and like these endogenous hormones, activates the intestinal guanylate cyclase C (GUCY2C) receptor, triggering the conversion of GTP to cGMP. The ensuing elevation of cGMP stimulates protein kinase G (PKG) and the cystic fibrosis transmembrane conductance regulator (CFTR), leading to excessive electrolyte and water secretion into the gut lumen and causing secretory diarrhea [68]. Others have confirmed the existence of a GUCA2A/2B–GUCY2C axis in intestinal cells and reported that ST activates GUCY2C by mimicking GUCA2A/B, leading to cGMP overproduction and disruption of the Wnt signaling pathway via interference with Frizzled-7 (FZD7) [69]. The basolateral efflux of cGMP is mediated by multidrug resistance protein 5 (MRP5), whereas intracellular cGMP is degraded by phosphodiesterase 5 (PDE5) [70]. In contrast, LT binds to ganglioside GM1 on the cell surface and activates CFTR, increasing intracellular cAMP and inhibiting Na^+^/H^+^ exchangers (NHEs) [70].

Baicalin was reported to mitigate ETEC-induced diarrhea in piglets by preventing bacterial adhesion to gut epithelial cells and reducing the ETEC-induced inflammatory response [71]. The electron-rich carboxyl and carbonyl groups of baicalin also facilitate the chelation of metal ions, which helps protect piglet intestinal cells, maintain epithelial barrier function, and preserve immune function, thereby protecting against diarrhea [72]. A baicalin–aluminum complex was found to decrease ETEC adherence to IPEC-1 porcine epithelial cells and to reduce CFTR mRNA expression levels as well as cAMP and cGMP generation, thereby blocking activation of the cAMP/cGMP–CFTR signaling cascade, but without significant inherent cytotoxicity [73]. In addition, treatment with this baicalin–aluminum complex increased NHE4 mRNA levels in infected IPEC-1 cells, which could mitigate ECET-induced disruptions in ion balance [73], although the mechanism underlying this effect remains unknown.

#### 4.1.2. Treatment of *Salmonella* Infections

*Salmonella* is a Gram-negative bacterium that readily forms biofilms on food-contact surfaces, and thus is a major cause of severe food poisoning [74]. Biofilm formation enhances the survival and resistance of *Salmonella* to antimicrobials, which is a key factor in outbreaks of foodborne gastrointestinal illnesses [74]. Thus, the development of effective antibiofilm strategies to combat *Salmonella*-caused diseases is particularly urgent [74]. Baicalin and carvacrol, a terpene found in spices such as oregano, have attracted attention as promising antimicrobial and antibiofilm agents [16,75,76]. Indeed, the combination was found to have synergistic antimicrobial activity against *Salmonella* [77] by damaging biofilm structures and reducing bacterial cell viability as revealed by confocal laser scanning and field-emission electron microscopy [77]. Furthermore, the same study reported that QS, virulence, and stress-response genes were dramatically downregulated by the combination of baicalin and carvacrol, suggesting that both compounds interfere with bacterial communication (QS) and virulence pathways [77]. Baicalin has also been shown to increase the efficacy of the Gram-negative antibiotic polymyxin against *Salmonella* [78]. In addition, baicalin was found to reverse the colistin resistance of all *mcr-1*-positive *Salmonella* strains tested (from 4–64 to 1/4–1/512 mg/L, an average 1736-fold reduction in the minimum inhibitory concentration [MIC]) when used in combination with the divalent cation chelator EDTA [78]. Molecular docking simulations and RT-PCR analysis also revealed that the baicalin–EDTA combination downregulated *mcr-1* gene expression and acted as a potent inhibitor of the MCR-1 resistance protein [78]. Detailed mechanistic studies further suggested that the baicalin–EDTA combination promoted oxidative damage to *Salmonella* by increasing tricarboxylic acid cycle activity and blocking bacterial antioxidant defenses as well as by downregulating genes associated with lipopolysaccharide stimulation and multidrug efflux pumps, thereby restoring the sensitivity of *Salmonella* to colistin [78]. Combined treatment with colistin, baicalin, and EDTA also significantly lowered bacterial burdens in the liver and spleen of infected animals compared with single or dual treatments [78]. Thus, the baicalin–EDTA combination shows promise as a nonantibiotic colistin adjuvant to treat *Salmonella* infections. However, further clinical trials are needed to validate its synergistic efficacy with colistin in vivo [78].

#### 4.1.3. Treatment of *Clostridioides difficile* Infections

*Clostridioides difficile* is an anaerobic spore-forming bacterium responsible for toxin-mediated human colitis [79]. In the USA, *C. difficile* infection (CDI) affects over 453,000 people annually and leads to ~29,000 deaths [80], making it a significant public health threat. Individuals on long-term antibiotic therapy are particularly susceptible to CDI [81]. Broad-spectrum antibiotic use disrupts the intestinal microbiota (induces dysbiosis), promoting *C. difficile* colonization and the production of its two main toxins, enterotoxin A (TcdA) and cytotoxin B (TcdB) [82]. These toxins act as glucosyltransferases, inactivating Rho family GTPases that regulate the host cell cytoskeleton. This effect compromises F-actin dynamics and the intracellular processing of tight-junction proteins (E-cadherin, ZO-1, occludin) in colonic epithelial cells, leading to the mislocalization of tight junctions and a weakened epithelial barrier [83]. The resulting barrier damage causes severe diarrhea and purulent inflammation, which can progress to pseudomembranous colitis or toxic megacolon [83].

Currently, antibiotics remain the first-line treatment for CDI. However, new treatment approaches are needed due to the emergence of highly infectious, antibiotic-resistant *C. difficile* strains [84]. Antivirulence therapy is gaining attention as a promising strategy to control antibiotic-resistant infections [84]. Instead of killing the pathogen or inhibiting its growth, attenuation of bacterial virulence reduces the selection pressure for resistance and minimizes effects on beneficial microbiota. Orally administered baicalin was reported to promote the growth of *Lactobacillus* and help maintain a more stable gut microbiota in mice (i.e., preventing dysbiosis) [85]. Furthermore, baicalin was reported to significantly downregulate the expression of the virulence genes *tcdA* and *tcdB* in *C. difficile* strain ATCC BAA 1870 by ~6.03- and 8.33-fold, respectively, compared with untreated controls, leading to effective inhibition of toxin production [86]. Baicalin also significantly downregulated sporulation-related genes *Spo0A*, *SigH*, and *CD2492* by 3.01-, 2.05-, and 3.69-fold, respectively, compared with the control in the same *C. difficile* strain, effectively inhibiting spore formation and growth [86]. These results indicate that baicalin can suppress *C. difficile* toxin production and spore development by targeting the transcriptional regulation of key virulence and sporulation genes, potentially reducing CDI severity, transmission, and relapse. Baicalin also markedly decreased the incidence of diarrhea, the severity of CDI symptoms, and intestinal damage in a mouse model [87], but did not adversely affect the overall diversity of the gut microbiota; rather, treatment increased the abundance of beneficial bacteria such as *Lachnospiraceae* and *Akkermansia muciniphila* and favorably modulated the gut microbiome composition [87]. Nonetheless, additional clinical and mechanistic research is required to confirm and extend these findings to human patients and to fully establish the efficacy of baicalin for managing CDI.

#### 4.1.4. Management of *Helicobacter pylori* Infection

*Helicobacter pylori* is a Gram-negative bacterium that can survive in the acidic environment of the stomach and induces gut pathology through production of virulence factors such as the cytotoxin-associated gene A (*cagA*) and vacuolating cytotoxin gene A (*vacA*) products (CagA and VacA). Colonization of the gastric mucosa by *H. pylori* can lead to chronic gastritis, gastric ulcers, and even gastric cancer [88,89]. During infection, the *H. pylori* CagA protein is delivered into host epithelial cells via a Type IV secretion system (T4SS) [90], which in turn triggers host expression of NF-κB-inducing kinase (NIK), gastrin, and homologs of the JHP0290 protein [91], and further activates downstream IκB kinase (IKK), protein kinase C (PKC), and secreted alkaline phosphatase signaling cascades, leading to activation of the NF-κB signaling pathway and the host immune response [92,93].

Conventional therapy for *H. pylori* infection involves one or two antibiotics plus a proton pump inhibitor and a bismuth compound [88]. However, antibiotic resistance of *H. pylori*, the acidic gastric environment, and patient nonadherence (often due to side effects) limit the efficacy of this regimen [94]. Therefore, it is imperative to design treatments that are both more effective and tolerable by patients. The gut microbiota structure can affect *H. pylori* proliferation and the severity of associated disorders [95]. Accordingly, the consumption of probiotics and prebiotics is an effective strategy to prevent *H. pylori* infection [96]. Baicalin was documented to inhibit *H. pylori* growth and promote the growth of beneficial gut microbes (probiotics), so it could potentially serve as a prebiotic or adjunct therapy for *H. pylori* infection [96]. In addition, baicalin has been shown to directly inhibit *H. pylori* growth by downregulating the *hefA* gene involved in multidrug efflux and the *vacA* gene encoding a major exotoxin that contributes to gastric cell adhesion. Baicalin has also been reported to suppress urease activity, thereby enhancing bacterial antibiotic susceptibility [96], and to stimulate the growth of the probiotic strain *Lactobacillus casei* in vitro. Chen et al. [97] observed that oral baicalin downregulated *vacA* expression, disrupted *H. pylori* adhesion and invasion into gastric cells, and lowered the levels of the proinflammatory cytokine interleukin (IL)-8 in infected mice. Also, 312.5 μM baicalin effectively inhibited *H. pylori* growth in the mouse stomach. Baicalin-treated animals further showed reduced serum IL-1β levels as well as lower titers of *H. pylori*-specific IgM and IgA. A synergistic therapeutic effect on *H. pylori* eradication was noted when baicalin was combined with *Lactobacillus rhamnosus* JB3 [97]. Therefore, baicalin may treat *H. pylori* infection through two complementary mechanisms, promoting the growth of beneficial gut flora and directly suppressing *H. pylori* growth. Combining probiotics with baicalin may improve the therapeutic outcomes for *H. pylori* infection while reducing the side effects and resistance risk associated with traditional antibiotic therapy [97].

### 4.2. Therapeutic Effects of Baicalin Against Bacterial Meningitis

Bacterial meningitis is among the most common central nervous system (CNS) infections. Although advances in medical technology and antibiotic use have significantly reduced the rate of meningitis-related mortality, it remains a major cause of neurological complications, especially in developing countries, where the incidence of long-term sequelae is roughly 2–4-fold greater than in developed countries (40–55% vs. 10–20%) [98]. Inflammatory responses in bacterial meningitis are driven by the components of bacterial cell walls and virulence factors that enter the subarachnoid space [99,100]. These bacterial antigens activate endogenous CNS immune cells, including monocytes/macrophages, microglia, astrocytes, and brain vascular endothelial cells, inducing the release of proinflammatory mediators such as tumor necrosis factor-α (TNF-α) and IL-1 [101]. These factors in turn increase the permeability of the blood–brain barrier (BBB) and further activate and recruit white blood cells, leading to white blood cell infiltration into the brain parenchyma and protein leakage into the cerebrospinal fluid (CSF) [101]. These infiltrating leukocytes then produce additional proinflammatory cytokines, amplifying the initial local inflammatory response, and upregulating tissue factor expression, inducing vascular thrombosis. The resulting exudation and inflammation reduce cerebral blood perfusion and increase intracranial pressure [101]. In severe cases, worsening meningeal and brain inflammation from excessive TNF-α and IL-1 production can cause irreversible neurocellular damage and necrosis, and these effects may be augmented by antibiotics. Therefore, appropriate adjunct therapy is crucial. Dexamethasone is often given alongside antibiotics such as ampicillin to dampen inflammation, but evidence suggests that dexamethasone may worsen neuronal injury in the hippocampus [102], underscoring the need to explore innovative adjunctive treatments for bacterial meningitis.

Baicalin has demonstrated anti-inflammatory effects by downregulating proinflammatory mediators such as monocyte chemoattractant protein-1 (MCP-1), cyclooxygenase, lipoxygenase, and inducible nitric oxide synthase (iNOS) during the early phase of the inflammatory response. These proteins are key to white blood cell recruitment and further cytokine synthesis that amplifies the local immune response [103]. Baicalin also reduces chemokine-induced cell migration by inhibiting chemokine binding to leukocytes expressing specific chemokine receptors [104]. Recent studies have reported that baicalin combined with ampicillin has significant therapeutic efficacy against *E. coli*-induced meningitis. Compared with ampicillin alone, this combination therapy significantly reduced CSF white blood cell count (4078 ± 450 × 10^6^/L vs. 4565 ± 273 × 10^6^/L), CSF protein concentration (1.89 ± 0.08 g/L vs. 2.70 ± 0.10 g/L), TNF-α (254 ± 7 pg/mL vs. 318 ± 12 pg/mL), IL-1 (344 ± 10 pg/mL vs. 414 ± 6 pg/mL), and lactate (5.1 ± 0.38 mmol/L vs. 5.70 ± 0.45 mmol/L) at 19 h post-infection [105]. The combination also significantly improved the intracranial pressure (12.5 ± 0.5 cmH_2_O), mean arterial pressure (76.1 ± 4.1 mmHg), and brain water content (80.99 ± 0.17%) compared with the other groups [105]. Notably, ampicillin monotherapy was found to exacerbate the rise in CSF whitelung blood cell count and protein, whereas baicalin co-administration counteracted this side effect [105]. By inhibiting inflammatory cytokine release, reducing intracranial hypertension and brain edema, and alleviating hypoxic-ischemic brain damage, baicalin could serve as an effective adjuvant that offsets the exacerbated inflammation and pathophysiological damage caused by early antibiotic use [105].

### 4.3. Therapeutic Effects for Lung Diseases

#### 4.3.1. Treatment of Lung Infections

Pneumonia resulting from lung infections remains a leading cause of death throughout the world, particularly in regions of endemic poverty. The lung epithelial surface is directly exposed to environmental microbes, and unsanitary conditions along with unhealthy lifestyle choices (e.g., smoking) increase the susceptibility to infection [106].

Chronic obstructive pulmonary disease (COPD) is characterized by airway remodeling and progressive inflammation, with cigarette smoke (CS) being the primary trigger. Plasminogen activator inhibitor-1 (PAI-1), a key regulator of fibrosis, has been found to drive COPD progression by promoting the expression of proinflammatory cytokines like TNF-α and IL-6 in the lungs [107]. Zhang et al. found that baicalin ameliorated CS-induced inflammatory cell infiltration in the rat airway and concomitantly reduced PAI-1 expression by airway cells both in vivo and in vitro [108]. Also, enzyme-linked immunosorbent assays confirmed that baicalin significantly lowered serum TNF-α and IL-1β levels in both CS-exposed rats and CS extract (CSE)-treated lung epithelial cells [108]. Mechanistic studies further suggested that baicalin inhibits PAI-1 through the upregulation of histone deacetylase 2 (HDAC2) and ensuing suppression of the PAI-1 expression regulator NF-κB as these effects were reversed by an HDAC2 inhibitor [108]. In another study, combined treatment with tobramycin (30 mg/kg) and baicalin hydrate (2 mg/kg) significantly reduced the pulmonary load of *Burkholderia cenocepacia* in mice (559 ± 119 CFU/g) compared with tobramycin treatment alone (4560 ± 1714 CFU/g) [109]. Furthermore, baicalin suppressed the pulmonary microbial imbalance in streptozotocin-induced diabetic mice and aided in reducing acute lung injury (ALI) caused by avian pathogenic *E. coli* through NF-κB pathway suppression [110].

Tuberculosis, a contagious disease caused by *Mycobacterium tuberculosis* (Mtb), is responsible for roughly 6% of global deaths and is increasing. One study found that treating Mtb-infected RAW 264.7 macrophages with 100 μM baicalin for 180 min significantly suppressed the PI3K/Akt/mTOR signaling pathway as evidenced by a 50% reduction in phosphorylated Akt and mTOR levels compared with untreated infected cells, and concurrently inhibited proinflammatory NF-κB pathway activation and reduced p65 phosphorylation by 42% [111]. Immunofluorescence also confirmed that baicalin treatment reduced nuclear p65 as evidenced by ~60% fewer nuclear p65-positive signals and also reduced inflammasome assembly as indicated by a 55% reduction in ASC-positive puncta compared with infected controls [111]. Western blotting further revealed reduced NLRP3 inflammasome protein (by 38%) and mature IL-1β (by 72%) in culture supernatant. These results suggested that baicalin inhibits Mtb-induced inflammatory responses via a dual mechanism, suppression of PI3K/Akt/mTOR pathway signaling and ensuing autophagic degradation of NLRP3 inflammasome components, leading to reduced IL-1β release, and inhibition of PI3K/Akt/NF-κB, leading to downregulation of Nlrp3 and pro-Il1b transcription and thereby blocking the priming step of inflammasome activation [111]. This dual regulation was also associated with enhanced macrophage clearance of Mtb and alleviation of tissue damage. Another study found that baicalin suppressed NLRP3 inflammasome activation and downregulated thioredoxin-interacting protein (TXNIP) expression by blocking the eIF2α and PERK pathways, potentially preventing the heat-induced programmed cell death (pyroptosis) of Mtb-infected macrophages [112].

#### 4.3.2. Therapeutic Effects on LPS-Induced Airway Inflammation

The outer membrane of Gram-negative bacteria contains lipopolysaccharide (LPS), a potent activator of the Toll-like receptor 4 (TLR4) pathway that strongly stimulates the human innate immune system [113]. During severe infections, excessive TLR4 stimulation can lead to severe inflammatory reactions [114]. For instance, LPS administration can damage pulmonary microvascular endothelial cells, increase alveolar–capillary barrier permeability, and induce pulmonary edema, refractory hypoxemia, pulmonary hypertension, and other severe life-threatening symptoms [115]. In 2008, Huang et al. reported that baicalin alleviated LPS-induced ALI in a rat model as evidenced by reduced lactate dehydrogenase (LDH) activity (a cell death marker), neutrophil infiltration, and protein content in bronchoalveolar lavage fluid (BALF) [116]. At 16 h post-LPS challenge, BALF protein was significantly greater compared to controls (~1500 mg/L vs. ~500 mg/L), indicating a major increase in alveolar–capillary permeability, while LDH activity in BALF was roughly double in the LPS group compared with controls (~100 vs. ~40 mAbs/min), reflecting aggravated lung cell injury [116]. In the 20 mg/kg baicalin treatment group, however, LDH activity was only ~50% greater (~60 mAbs/min), indicating substantial cytoprotection [116]. The LPS-challenged lungs also exhibited typical ALI features, including massive neutrophil infiltration (~195 per high-power field vs. ~28 in controls), thickened alveolar walls, perivascular edema, and alveolar hemorrhage, while baicalin pre- or posttreatment significantly reduced neutrophil infiltration (from ~88 to ~81 per field), indicating attenuation of pulmonary inflammation [116]. In 2016, Ding et al. similarly reported that baicalin alleviated LPS-induced ALI in mice, and further revealed that this effect was associated with inhibition of the CX3CL1–CX3CR1 axis and NF-κB pathway, as well as with reduced Akt phosphorylation [117]. Specifically, 200 mg/kg baicalin pretreatment reduced CX3CL1 and CX3CR1 expression in LPS-challenged wild-type mice by 47% and 53%, respectively, reduced eosinophil and other inflammatory cell counts in BALF by 40–60%, lowered phosphorylated NF-κB and IκBα levels by ~49–58%, serum proinflammatory cytokines (like TNF-α) by ~51–61%, and phospho-Akt by ~55%, while normalizing the lung wet/dry weight ratio from 6.3 ± 0.3 to 4.9 ± 0.2 and reducing lung pathology score from 4.2 ± 0.5 to 1.8 ± 0.3 [117]. Baicalin still decreased CX3CR1 expression by 62%, phosphorylated AKT levels by 48%, and the lung wet-to-dry weight ratio by 28% in CX3CL1-knockout mice, confirming the role of CX3CL1 in mediating LPS-induced ALI pathology and indicating that baicalin’s protective effects are at least partially dependent on the CX3CL1-CX3CR1 axis [117]. These inhibitory effects of baicalin were also dose-dependent; at 200 mg/kg, p-Akt and p-NF-κB levels were reduced by 55% and 58%, respectively, compared with 21% and 25% at 50 mg/kg [117]. Moreover, Duan et al. reported that baicalin upregulated miR-200b-3p, resulting in the inhibition of inflammatory proteins such as iNOS, NF-κB p65, phospho-ERK1/2, and phospho-JNK1, and alleviating LPS-induced inflammation in alveolar type II cells (presumably by blocking the ERK/JNK pathways) [118]. Meng et al. also reported that baicalin activated the Nrf2/HO-1 antioxidant pathway, and significantly reduced inflammatory cell infiltration, proinflammatory mediator levels, and oxidative product synthesis while restoring antioxidant enzyme activity in lung tissue [119]. Furthermore, baicalin has been reported to augment the pulmonary protection conferred by traditional drugs. For example, Zhang et al. found that combined baicalin–magnesium treatment effectively reduced LPS-induced inflammatory responses and oxidative stress, and inhibited the TLR4/NF-κB pathway, thereby reducing ALI [120].

### 4.4. The Therapeutic Effect of Baicalin on Sepsis

Sepsis is a severe condition arising from dysregulated host responses to infection, leading to organ dysfunction and failure. One common cause of sepsis is infection by *Staphylococcus aureus*—particularly methicillin-resistant *Staphylococcus aureus* (MRSA)—which poses a major treatment challenge due to multidrug resistance and is associated with poor prognoses and high mortality [121,122]. Thus, the development of novel strategies is urgently needed to combat MRSA infections. Host-directed therapy (HDT) is a new treatment approach that focuses on modulating the host’s immune response rather than directly targeting the pathogen [123]. In the initial stage of infection, an appropriate inflammatory response helps control the infection. Innate immune cells such as dendritic cells (DCs) and macrophages are activated by bacterial signals and release chemokines that recruit neutrophils and monocytes to the infection site. Monocytes then differentiate into macrophages and, together with neutrophils, phagocytose bacteria to prevent dissemination. Macrophages and DCs also function as antigen-presenting cells, processing bacteria and presenting antigens to activate T and B cells, which eliminate the pathogen [124]. However, in severe infections, the innate response can become hyperactive, producing excessive cytokines that trigger a “cytokine storm,” leading to massive neutrophil infiltration into organs, complement activation, coagulation dysfunction, and ultimately systemic inflammatory response syndrome. Therefore, controlling the inflammatory response is crucial for improving sepsis survival.

Baicalin is a promising candidate for HDT strategies against severe sepsis caused by antibiotic-resistant bacteria, including MRSA [125]. Baicalin significantly inhibited the production of proinflammatory cytokines (like IL-6 and TNF-α) in primary peritoneal macrophages, immortalized bone marrow-derived macrophages (iBMDMs), and DCs stimulated by the TLR2 agonist Pam3CSK4, heat-killed MRSA, or peptidoglycan (PGN). For instance, 100–200 μM baicalin reduced IL-6 by 40–60% and TNF-α by 35–50% in macrophages stimulated with Pam3CSK4 [125], while 200 μM baicalin decreased IL-6 and TNF-α secretion from HK-MRSA-stimulated iBMDMs by over 60%. Additionally, 100–200 μM baicalin suppressed IL-6 and TNF-α expression in PGN-stimulated macrophages by 30–45% [125]. Baicalin also dose-dependently inhibited TLR2-mediated activation of the ERK/JNK MAPK and NF-κB pathways, as evidenced by a 50–70% reduction in the phosphorylation of ERK/MAPK molecules and ~40% reduction in NF-κB luciferase reporter activity in RAW 264.7 cells [125]. In MRSA-infected mice (2 × 10^8^ CFU intraperitoneally) as well, baicalin monotherapy (100 mg/kg) reduced serum TNF-α by ~35% and increased levels of the immunomodulatory cytokine IL-10 by 4.4-fold compared with untreated mice [125]. Baicalin also acted synergistically with vancomycin to reduce serum TNF-α by ~60%, elevate IL-10 5.2-fold, and reduce MCP-1 and IFN-γ by 20–30% versus monotherapy. The combination of baicalin and vancomycin also reduced bacterial loads in the liver and kidney by ~1.5 log_10_ and ~1.2 log_10_ CFU/g, respectively, and the histological injury scores in these organs by half relative to MRSA infection alone [125].

Baicalin was also reported to treat polymicrobial sepsis induced by cecal ligation and puncture (CLP) in a C57/Bl6 mouse model [126]. Baicalin improved bacterial clearance and extended survival in septic mice, reduced neutrophils in peritoneal lavage fluid, alleviated neutrophil infiltration and histological damage in the lungs and liver, decreased proinflammatory cytokines (e.g., IL-17, IL-6, TNF-α) in the blood, increased anti-inflammatory IL-10 in blood, and inhibited apoptosis of CD3^+^ T cells in the thymus. Compared with CLP alone, baicalin-treated mice had higher counts of CD4^+^ and CD8^+^ T cells and DCs and lower counts of CD4^+^CD25^+^ regulatory T cells [126]. In summary, baicalin can prolong the survival of polymicrobial sepsis model mice through its antibacterial, anti-inflammatory, and antiapoptotic effects [126]. However, the precise mechanisms mediating the anti-inflammatory and antiapoptotic effects of baicalin need further investigation, and its efficacy in human sepsis patients remains to be validated.

**Table 3:** Therapeutic efficacy of baicalin against bacterial diseases.

Disease	Bacteria	Experimental System	Drug Concentration	Effect	Mechanisms	Reference
Gastrointestinal Diseases	*Escherichia coli*	In vivo	Baicalin: 100 and 500 mg/kg	Improved growth performance; repaired intestinal morphology; regulated intestinal flora (mitigated dysbiosis); alleviated inflammation	Promoted beneficial bacterial colonization, inhibited pathogen growth; reduced accumulation of inflammatory metabolites, increased phospholipids/amino acids; regulated Th17/Treg balance and the IL-17 pathway; restored immune homeostasis	[71]
	ETEC	In vitro	Baicalin complex: 0–100 μg/mL	Inhibited ETEC adhesion to IPEC-1 cells; reduced ETEC-induced intracellular cAMP/cGMP; decreased CFTR mRNA, increased NHE4 mRNA	Weakened ETEC adhesion to IPEC-1 cells; inhibited cAMP/cGMP–CFTR pathway activity; regulated gene expression	[73]
	*Salmonella enterica ser. Typhimurium*	In vitro	Baicalin plus carvacrol, ratio set according to MIC	Exhibited strong antibacterial activity; no effect on chicken color/quality; destroyed biofilms, reduced pathogen viability; downregulated quorum sensing, virulence, and stress genes	Baicalin interfered with quorum sensing, inhibited synthesis, and destroyed bacterial membranes; carvacrol disturbed bacterial membranes increased permeability/depolarization; the combination targeted communication/virulence and demonstrated multifaceted antibacterial effects	[77]
	*Salmonella*	In vitro	Baicalin (1250 mg/L); EDTA (125 mg/L or 62.5 mg/L); colistin (2–32 mg/L), alone or in combination.	Baicalin and EDTA alone or in combination enhanced colistin activity and reversed drug resistance of all *Salmonella* strains; the three-drug combination significantly reduced bacterial load in the liver and spleen of infected mice	Baicalin bound MCR-1 and EDTA chelated zinc ions, thereby jointly inhibiting MCR-1 activity; baicalin enhanced the effect of colistin through multiple pathways such as accelerating the tricarboxylic acid cycle	[78]
		In vivo	Colistin (20 mg/kg) + Baicalin (50 mg/kg) + EDTA (10 mg/kg).			
	*Clostridium difficile*	In vitro	Baicalin: 700 μg/mL	Reduced *Clostridium difficile* toxin damage to Vero cells (cytotoxicity ↓~85%); spore count ↓1.1–1.3 log_10_ in 72 h, fully inhibiting proliferation	Downregulated *tcdA/tcdB*/*tcdR*, *Spo0A*/*SigH*, and *fbp64*/*cwp84*	[86]
	*Clostridioides difficile*	In vivo	Baicalin: 250 and 500 mg/kg	Reduced diarrhea rate/clinical score, promoted weight recovery; lowered the colonic pathology score, alleviated damage/inflammation; increased beneficial bacteria, reduced pathogens; reduced fecal spores, decreased toxin cytotoxicity	Inhibited the production of *Clostridium difficile* toxin and spore germination; regulated intestinal flora balance and enhanced colonization; suppressed inflammation and diarrhea; alleviated intestinal damage	[87]
	*Helicobacter pylori*	In vitro	Baicalin: 1.00 mg/mL	The strongest activity against *Helicobacter pylori*, with an inhibition zone of 18.90 mm; promoted the growth of *Lactobacillus casei* and slightly promoted the growth of *Lactobacillus brevis*	Inhibited *hefA*/*VacA* (reduced resistance/virulence) and urease (interfered with gastric survival); blocked adhesion/invasion; promoted *Lactobacillus casei*, did not inhibit probiotics; bound transcription proteins (blocked RNA synthesis), target transporters, acted on drug-resistant bacteria	[96]
	*Helicobacter pylori*	In vitro	Baicalin alone: 80 μM, 156.25 μM, 312.5 μM; combined with *Lactobacillus rhamnosus* JB3: 80 μM.	High baicalin (312.5 μM) reduced bacterial load, alleviated infection; 80 μM + LR-JB3 synergistically killed bacteria; reduced gastric inflammation/damage, inhibited *vacA*; no probiotic inhibition, maintained flora balance, mitigated antibiotic side effects	Inhibited *hefA* (increased antibiotic sensitivity) and urease; downregulated *vacA* (reduce toxin), inhibited adhesion/invasion; reduced IL-8/IL-1β, IgA/IgM (alleviated inflammation/immune damage); with LR-JB3, synergistically inhibited *Helicobacter pylori*	[97]
		In vivo	0–250 uM			
Bacterial Meningitis	LPS	In vitro	Baicalin: 10, 25, 50 μM	Significantly reduced LPS-induced release of cytokines/chemokines (IL-6, G-CSF, etc.); inhibited NO, ROS, and intracellular calcium accumulation; downregulated inflammation-related genes; decreased phosphorylated p38 MAPK and Fas	Inhibited intracellular calcium release via the calcium-CHOP pathway; downregulated inflammatory-related genes; reduced p38 MAPK phosphorylation and inflammatory mediator production	[104]
	*Escherichia coli*	In vivo	Baicalin: 20 mg/kg	Mitigated ampicillin-aggravated increases in cerebrospinal fluid (CSF) white blood cells and protein; reduced CSF TNF-α/IL-1 to alleviate inflammation; decreased intracranial pressure (longer-lasting than mannitol) and mean arterial pressure; lowered CSF lactic acid and brain water content	Inhibited inflammatory genes and monocyte chemoattractant protein-1 (thereby reducing leukocyte recruitment, cytokine production, and chemokine binding); reduced TNF-α/IL-1/inflammatory mediators (thereby alleviating inflammation, cerebral edema, and brain damage); acted synergistically on *E. coli* when combined with ampicillin	[105]
Chronic Pneumonia	*Mycobacterium tuberculosis* (Mtb)	In vitro	Baicalin: 100 μM	Significantly enhanced macrophage clearance of intracellular Mtb (86.7% bactericidal rate); reduced Mtb-induced ASC speck formation, inhibited NLRP3 inflammasome activation and IL-1β secretion; effectively downregulated PI3K/Akt/mTOR and NF-κB pathway activation	Inhibited PI3K/Akt/mTOR pathway, activated macrophage autophagy (promoted LC3-II formation, p62 degradation); inhibited NLRP3 inflammasome activation (reduced component expression/interaction, mature IL-1β); inhibited NF-κB (reduced p65 phosphorylation/nuclear translocation); promoted autophagosome-inflammasome co-localization (indicating autophagic degradation of components, indirect anti-inflammatory effect)	[111]
Tuberculosis	*Mycobacterium tuberculosis* (Mtb)	In vitro	Baicalin: 100 μM	Alleviated Mtb-induced macrophage pyroptosis (reduced GSDMD-N, LDH, and cell death); inhibited inflammation (lower IL-1β, HMGB1, and NLRP3 components); regulated endoplasmic reticulum stress (reduced BIP, CHOP, and inhibited PERK/eIF2α); blocked TXNIP–NLRP3 interaction to inhibit inflammasomes	Inhibited the PERK/TXNIP/NLRP3 axis (downregulated PERK/eIF2α phosphorylation, reduced ER stress proteins); decreased TXNIP and its binding to NLRP3; reduced the NLRP3–ASC–pro-caspase-1 interaction (thereby blocking inflammasome assembly); inhibited caspase-1-mediated IL-1β expression, GSDMD cleavage, and pyroptosis	[112]
	LPS	In vitro	Baicalin: 50, 75 μM	Significantly reduced LPS-induced A549 damage and IL-6 expression; reduced apoptosis, improved lung epithelial morphology; downregulated FSTL1 to inhibit inflammation/damage	Upregulated miR-200b-3p; inhibited ERK/JNK activity (reduced p-ERK1/2, p-JNK) and NF-κB activity (resulting in lower iNOS, COX-2); suppressed LPS-induced inflammation	[118]
	LPS	In vivo (gavage administration)	Baicalin: 200 mg/kg	Significantly improved LPS-induced mouse acute lung injury (reduced lung injury score); reduced inflammatory cell infiltration in BALF and blood; lowered BALF total protein and TNF-α levels; restored SOD/CAT activity, reduced lipid peroxidation (MDA), and alleviated alveolar structure destruction	Activated the Nrf2-mediated HO-1 pathway; upregulated nuclear Nrf2 and cytoplasmic HO-1 in lung tissue, enhanced SOD/CAT activity, reduced MDA; inhibited LPS-induced TNF-α/IL-6/IL-1β release, alleviated oxidative stress and inflammation	[119]
Sepsis	MRSA	In vitro	Baicalin (50–200 μM);	Reduced the secretion of proinflammatory factors such as IL-6 and TNF-α while increasing IL-10 levels; decreased bacterial load in the liver and kidney, alleviated inflammatory cell infiltration and necrosis, and improved pathological scores; reduced the mortality of MRSA-infected mice when used alone or in combination with vancomycin/dexamethasone	Inhibited ERK/JNK MAPK and NF-κB activation, reduced proinflammatory factors; enhanced bacterial clearance, synergistically protected organs, and alleviated excessive inflammation when combined with vancomycin	[125]
		In vivo (intraperitoneal injection)	Baicalin (100 mg/kg)			
	Multiple microorganisms (from the intestinal flora)	In vivo	Baicalin: 100 mg/kg	Significantly improved the 8-day survival rate of CLP-septic mice; alleviated organ damage (e.g., lung, liver) and reduced damage scores; decreased bacterial counts and neutrophil infiltration in the blood and abdominal cavity; regulated cytokine balance, inhibited lymphocyte apoptosis, improved immune cell distribution, and enhanced anti-infection and immune regulation capabilities	Enhanced bacterial clearance and reduced bacterial load; inhibited excessive inflammation, reduced abdominal neutrophil infiltration and proinflammatory factors (e.g., TNF-α), while increasing anti-inflammatory factor IL-10; inhibited thymic CD3^+^ T cell apoptosis, increased splenic CD4^+^, CD8^+^ T cells, and dendritic cells, reduced the proportion of regulatory T cells, thereby improving immune function	[126]

## 5. Efficacy of Baicalin in Combination with Other Bioactive Substances

Numerous studies have confirmed that baicalin can exert synergistic antibacterial effects when combined with various antibiotics [127]. Chen et al. found that the combined administration of baicalin and ampicillin had significant synergistic effects against MRSA and *Stenotrophomonas maltophilia* that were mediated by multiple mechanisms, including membrane disruption, cell wall synthesis inhibition, and resistance gene regulation. In an MRSA-infected mouse model, this combination reduced bacterial loads in the liver and kidney by ~1.5 log_10_ and ~1.2 log_10_ CFU/g, respectively, and decreased liver and kidney injury scores by 50–60% compared with MRSA-infected controls, approaching levels observed in uninfected tissues [128]. Luo et al. reported that baicalin combined with tobramycin enhanced the clearance of *P. aeruginosa* biofilms in a time-dependent manner. Over 24 h, the combination reduced biofilm biomass by 42.3% and viable cell count by 1.8 log_10_ compared to tobramycin alone, while over 96 h, the combination reduced biofilm biomass by 58.7% and viable count by 2.1 log_10_ [18]. Furthermore, baicalin enhanced the effect of azithromycin against multidrug-resistant *Staphylococcus saprophyticus* biofilms by upregulating the WalK/WalR two-component regulatory system genes (*walK*, *walR*, *yycI*, *yycH*) by 2–4 fold compared to azithromycin alone [17]. Therefore, baicalin holds excellent potential for combating biofilm-related bacterial diseases.

Baicalin has also demonstrated enhanced activity when combined with other plant-derived compounds. For instance, combining baicalin with osthole, a coumarin from Cnidium plants, alleviated *S. aureus*-induced damage to A549 lung cells and protected mice from *S. aureus* pneumonia [129]. Likewise, a combination of resveratrol, baicalin, and polymyxin demonstrated strong synergistic activity against *mcr-1*-positive *E. coli* in cell culture and animal models [130]. Mechanistic studies further suggested that this combination increases polymyxin-mediated membrane damage, disrupts the proton motive force, suppresses efflux pump activity, and reduces bacterial ATP production [130]. Molecular docking simulations also suggested that resveratrol and baicalin can stably bind to the MCR-1 protein, implying that combined use may effectively inhibit MCR-1. This combination could serve as a colistin adjuvant, providing a novel strategy for treating infections by *mcr-1*-positive *E. coli* [130].

## 6. Safety

Safety studies of baicalin are crucial for advancing its medical application. Numerous cell and animal model studies have defined dosage ranges for baicalin (alone and in some cases for co-administration with other agents) that do not impact essential non-target functions. Hao et al. found that 25–100 mg/kg baicalin alleviated pulmonary inflammation, cell apoptosis, and alveolar destruction in COPD models by upregulating HSP72 expression and inhibiting JNK pathway activation without inducing weight loss, liver/kidney dysfunction (as indicated by normal serum concentrations of alanine transaminase [ALT], aspartate transferase [AST], and other markers), or histological damage to lung, liver, and kidney tissues (as evidenced by hematoxylin and eosin staining) [131]. Feng et al. also reported that 50 μM baicalin alone did not reduce cardiomyocyte viability or activate the TLR4/NF-κB inflammatory pathway [132]. In rats, oral baicalin at 100 mg/kg did not affect weight gain or cardiac function as evidenced by stable serum levels of cardiac injury markers (NT-proBNP, BNP, cTnT, CK-MB) and inflammatory factors (CRP, LDH, MCP-1, TNF-α) compared to controls, while histology revealed normal myocardial morphology with no signs of inflammation or damage [132]. Baicalin has also demonstrated no detectable toxic reactions in several animal disease models, including allergic asthma and alcoholic liver disease models [133,134,135,136] (Table 4). In fact, no severe adverse acute reactions to baicalin have been reported to date, although chronic toxicity may occur with longer-term administration of higher doses. For example, Zhang et al. reported that 200 mg/kg baicalin given to rats for 28 days caused no abnormalities, while mild renal toxicity appeared in male rats after 58 days [120]. In addition, after 8 days of intragastric administration of high-dose baicalin (400, 800, and 1600 mg/kg/day), the levels of blood urea nitrogen and creatinine in the plasma of rats significantly increased, indicating impairments of renal filtration and excretion functions [137]. Masson staining also showed markedly increased collagen deposition in the renal tissues of rats receiving high-dose baicalin, suggesting renal fibrosis progression [137]. Thus, while generally safe for animals in therapeutic dose ranges, there is a potential risk of chronic renal toxicity with prolonged high dosing.

## 7. Future Challenges and Novel Strategies for the Treatment of Bacterial Infections Using Baicalin

In pharmacology, bioavailability refers to the extent and rate at which a drug is absorbed into the systemic circulation, typically expressed as the percentage of an oral dose [138]. Drug absorption efficiency is closely related to the drug’s permeability coefficient; generally, drugs with ~1% absorption have a permeability of ~1.0 × 10^−6^ cm/s, those with 1–100% absorption have a permeability of 1.0 × 10^−6^ to 0.1 cm/s, and those with <1% absorption have a permeability < 1.0 × 10^−7^ cm/s [139]. Baicalin is characterized by low water solubility (<0.1 mg/mL) and low permeability (*P*_app_ = 0.037 × 10^−6^ cm/s), which prevent it from easily crossing lipid bilayers via passive diffusion, leading to poor absorption and low oral bioavailability [140]. To improve the bioavailability of baicalin, researchers have developed various nanoformulations, prodrugs, and carrier complexes.

IMeher et al. constructed a polyethyleneimine (PEI)-modified heparin carbon dot nanosystem and reported increased fluorescence quantum yield from 2.9% to 9.4% via PEI surface passivation [141]. The positive charge of PEI also enabled electrostatic interactions with the hydroxyl groups of baicalin, improving water solubility and targeting. The nanosystem showed a high baicalin loading efficiency (94.4%) and pH-responsive release, significantly enhancing the bioavailability and toxicity against tumor cells [141]. Farouk et al. prepared baicalin nanoemulsions by spontaneous emulsification, which improved the dispersibility, intestinal permeability, and lymphatic transport of baicalin, thereby markedly increasing its bioavailability [141]. A low dose of this nanoemulsion (20 mg/kg) proved more effective at alleviating cisplatin-induced hepatotoxicity in mice than a higher dose (100 mg/kg) of free baicalin in suspension [142]. Wei et al. developed baicalin liposomes (BA-LP) using effervescent dispersion technology [7]. The key advantages of this formulation included increased drug contact surface area due to the nano-sized liposome structure (mean diameter ~373 nm) for improved solubility, enhanced intestinal mucosal permeability conferred by the phospholipid components, prolonged absorption time, and reduced first-pass metabolism due to sustained-release properties [7]. Compared with baicalin carboxymethylcellulose suspension (BA-CMC), BA-LP significantly improved the bioavailability of baicalin as evidenced by a 3.04-fold increase in the area under the concentration–time curve (AUC) compared to BA-CMC (37.64 mgh/L vs. 12.397 mgh/L), and a 2.82-fold increase in the peak concentration (C_max_) (3.52 mg/L vs. 1.25 mg/L for BA-CMC) [7]. This BA-LP formulation also altered the distribution of baicalin in vivo, with drug concentrations in the liver, kidney, and lung 5.59-, 2.33-, and 1.25-fold higher than attained by BA-CMC, respectively, thereby enhancing the therapeutic effects in target organs [7].

Currently, baicalin prodrug design is focused on improving water solubility, bioavailability, and other properties through molecular modification and self-assembly, thereby enhancing pharmacological activity. Yang et al. developed a functional polymer hydrogel ((AG-P_m_)-OC) based on baicalin for treating osteoarthritis. They first synthesized a polymer (Pm) by esterifying and RAFT-polymerizing baicalin with hydrophilic monomers and then formed a dynamically reversible hydrogel via Schiff-base bonding with aminated gelatin and oxidized chondroitin sulfate [24]. This hydrogel prolonged baicalin retention in the joint cavity (AUC increased 2–3 times) and was preferentially taken up by synovial fibroblasts [24]. By inhibiting the YAP1 signaling pathway, it reduced glycolysis and inflammatory factor secretion, regulated macrophage polarization, and provided enhanced anti-inflammatory, cartilage-protective, and analgesic effects in an osteoarthritis model [24]. Chang et al. developed a baicalin–resveratrol self-assembled nanoparticle (BRN) for the synergistic treatment of respiratory syncytial virus (RSV)-induced pneumonia [143]. Baicalin and resveratrol were coassembled via π–π stacking and hydrogen bonding. The half-maximal effective concentration (EC_50_ = 11.2 μM) of BRN was much lower than that of free baicalin (21.3 μM) or resveratrol (30.1 μM), and the selectivity index (SI = 44.1) was ~2.4 times higher than that of free baicalin (18.2), indicating greater antiviral efficacy and safety [143]. In vivo imaging showed that after nebulized inhalation, BRN fluorescence in the lungs remained significantly higher than that of a free baicalin–resveratrol mixture at 6 h, with the pulmonary AUC ~2–3 times larger [143]. In an RSV pneumonia mouse model, BRN reduced lung viral load by ~60% versus untreated controls, improved lung function (tidal volume and minute ventilation restored to >80% of normal), and alleviated inflammation by modulating M1/M2 macrophage polarization (increasing the M2 proportion by ~40%), outcomes significantly superior to free baicalin, free resveratrol, or a physical mixture free drugs [143].

In addition, introducing phospholipids to these formulations can enhance the dissolution and absorption of baicalin by improving its lipophilicity and hydrophilicity. Chen et al. (2018) mixed baicalin with soybean phospholipids at a 1:2 mass ratio, added tetrahydrofuran, refluxed at 55 °C for 1 h, and then prepared a baicalin–phospholipid complex (BAPC) by vacuum rotary evaporation at 50 °C [144]. In duck embryo hepatocyte cultures, these BAPCs demonstrated a significantly better inhibitory effect on duck hepatitis A virus type 1 (DHAV-1) than free baicalin alone as evidenced by RT-qPCR analyses of viral gene expression [144]. Moreover, BAPC simultaneously inhibited viral protein translation and RNA synthesis, whereas baicalin alone only inhibited protein translation, resulting in ~1.5–2 times higher antiviral activity for BAPC [144]. In a subsequent study, Chen et al. (2022) combined phospholipid complexation with nasal delivery to improve the bioavailability of baicalin [145]. They found that intranasal administration of 40 mg/mL *S. baicalensis* extract–phospholipid complex (BEPC) significantly reduced neurological deficit scores and cerebral infarct size in a middle cerebral artery occlusion rat model of stroke compared to baicalin extract alone and other BEPC administration routes, and this superior efficacy was associated with ~2–3 times greater bioavailability conferred by nasal delivery of this phospholipid complex [145]. Li et al. covalently attached baicalin to carboxylated multi-walled carbon nanotubes (MWCNTs) to form MWCNT-BAs and then coated these with folic acid-modified carboxymethyl chitosan (CMCS-FA), creating a core–shell structured CMCS-FA–MWCNT-BA complex with ~2–3-fold greater targeting of oral squamous cell carcinoma tumor cells due to folate receptor binding and greater solubility and biocompatibility conferred by CMCS compared to free baicalin [146]. These modifications represented promising strategies for the precise delivery of baicalin and other natural drugs to therapeutic sites.

## 8. Conclusions and Future Perspectives

Baicalin is a flavonoid compound extracted primarily from the dried roots of *Scutellaria baicalensis*, a plant in the Lamiaceae family. This key component of traditional Chinese medicine is gaining prominence in modern scientific research due to a plethora of natural sources, improved extraction techniques, multiple documented bioactivities, synergistic efficacies with various other natural compounds and pharmaceuticals, a promising safety profile, and advances in therapeutic delivery.

A review of extraction technologies indicated that, compared with traditional reflux extraction, emerging technologies such as UAE and MAE offer advantages of greater efficiency and environmental sustainability. However, these emerging technologies also have limitations, making it difficult to rely solely on any single method for baicalin extraction. It is noteworthy that due to the thermosensitivity of baicalin, its stability may be significantly reduced under high-temperature conditions. Thus, high temperatures generated during ultrasonic and microwave-assisted extractions may affect the stability of baicalin. Therefore, integrating different extraction techniques to leverage their respective strengths while addressing stability issues is of great importance for future development.

This article also explored the therapeutic potential of baicalin for treating various bacterial infectious diseases, including gastrointestinal infections, bacterial meningitis, pulmonary diseases, and sepsis. Studies have found that baicalin exhibits similar molecular mechanisms across these conditions, such as inhibiting bacterial adhesion, disrupting biofilms, suppressing the expression of resistance and efflux pump genes, and interfering with QS and virulence pathways, thereby reducing bacterial load and associated tissue damage. In addition, baicalin can inhibit inflammation-related pathways (e.g., PI3K/Akt/NF-κB, ERK/JNK, PERK/eIF2α), downregulate inflammatory factor expression, and reduce inflammation-induced cell apoptosis. However, most mechanistic studies of baicalin have been conducted in vitro or in animal models, so clinical efficacy and safety require much further investigation. Clinical trials are warranted (for example, a phase I dose-escalation trial in patients with sepsis) to evaluate the safety and pharmacokinetics of baicalin, perhaps starting with intravenous infusion of 50–200 mg/day. It is also necessary to identify potential target organs for toxicity through nonhuman primate studies and to complete carcinogenicity assessments and Investigational New Drug applications to meet regulatory requirements.

Furthermore, the clinical use of baicalin is currently limited by its poor solubility and low bioavailability. To address these problems, various nanotechnologies have been introduced. By loading baicalin into nanocarriers (such as nanoparticles and liposomes), solubility, stability, and bioavailability can be improved, enhancing therapeutic efficacy. In addition, given the antibacterial effects of baicalin, further studies of its in vitro activity and in vivo efficacy (e.g., in animal models) for complicated urinary tract infections are warranted. In the future, the clinical translation of baicalin will need to overcome bottlenecks such as the lack of clinical trial data, unclear toxicity profiles, and regulatory compliance hurdles. Meanwhile, it is essential to expand research into new application areas (such as urinary tract infections) and to promote the modern development of baicalin through technological integration and full-chain (bench-to-bedside) research.

## Figures and Tables

**Table 1:** Major sources of baicalin.

Plant	Tissue	Content	References
*Scutellaria* *baicalensis*	Root	26.05 ± 3.9 mg/g fresh weight; 84.21 mg/g dry weight	[4,26]
	Stem	0.02 mg/g fresh weight; 0.84 mg/g dry weight	[4,26]
	Leaf	0.24 ± 0.1 mg/g fresh weight; 1.49 mg/g dry weight	[4,26]
	Flower	3.86 mg/g dry weight	[26]
*S. lateriflora*	Root	1.48 ± 0.4 mg/g fresh weight; 14.91 mg/g dry weight	[4,28]
	Stem	0.27 ± 0.1 mg/g fresh weight 4.7 mg/g dry weight	[4,28]
	Leaf	11.66 ± 2.9 mg/g fresh weight; 33.58 mg/g dry weight	[4,28]
*S. arenicola*	Root	0.03 mg/g fresh weight	[4]
	Stem	0.01 mg/g fresh weight	
	Leaf	0.04 mg/g fresh weight	
*S. integrifolia*	Root	0.02 mg/g fresh weight	[4]
	Stem	0.01 mg/g fresh weight	
	Leaf	0.01 mg/g fresh weight	
*Siegesbeckia pubescens Makino* (Different regions in China)	Not clearly distinguished	Hebei sample 1: 0.14 mg/g; Hebei sample 2: 0.006 mg/g; Shenyang sample: 0.099 mg/g; Anhui sample: 0.082 mg/g	[29]
*V. teucrium*	Not clearly distinguished	0.347 mg/g dry weight	[30]
*V. jacquinii*	Not clearly distinguished	0.009 mg/g dry weight	[30]
*V. urticifolia*	Not clearly distinguished	0.779 mg/g dry weight	[30]
*O. indicum*	Seed	Seed extract: 68.7 ± 1.1 mg/g; orange-red crystals: 68.2 ± 0.4 mg/g; yellow precipitate: 16.2 ± 0.4 mg/g	[31]
	Young fruit	1.9 mg/g	
	Flower	0.4 mg/g	
*S. wrightii*	Root	122.14 ± 1.42 mg/g dry weigh	[32]
	Stem	0.92 ± 0.02 mg/g dry weight	
	Leaf	0.51 ± 0.07 mg/g dry weight	
*S. tomentosa*	Root	17.30 ± 0.22 mg/g dry weight	[32]
	Stem	10.63 ± 0.26 mg/g dry weight	
	Leaf	1.05 ± 0.03 mg/g dry weight	
*S. racemosa*	Root	11.11 ± 0.24 mg/g dry weight	[32]
	Stem	10.59 ± 0.11 mg/g dry weight	
	Leaf	15.21 ± 0.11 mg/g dry weight	
*S. baicalensis* (different sampling sites, abbreviations defined in text)	Root (CLY)	~160 mg/g	[33]
	Root (CDY)	~100 mg/g	
	Root (LHY)	~140 mg/g	
	Root (LHZ)	~150 mg/g	
	Root (WDZ)	~180 mg/g	
*S. baicalensis* (different light treatments)	Root (white light treatment for 2 weeks)	100.42 ± 0.32 mg/g dry weight	[34]
	Stem (red light treatment for 4 weeks)	0.17 ± 0.05 mg/g dry weight	
	Leaf (red light treatment for 4 weeks)	0.80 mg/g dry weight	

**Table 4:** Safety evaluation of baicalin in various pathological injury models.

Pathological Injury Model	Research Model	Administration Route	Dose/Treatment	Toxicity Indicators	Safety Evaluation	References
Chronic Obstructive Pulmonary Disease	C57BL/6 mice	Gavage	Baicalin 50, 100 mg/kg, once daily for 7 days	Body weight change; Pulmonary histopathology	No weight loss or obvious histopathological damage; no signs of abnormal liver or kidney function	[131]
	MLE-12 cells	In vitro	Baicalin 20 μmol/L, treated for 24 h	Cell viability; Cell morphology	No inhibition of cell viability, normal morphology; no cytotoxicity	
Doxorubicin-induced Cardiotoxicity	SD rats	Gavage	Baicalin 100 mg/kg/d for 6 weeks	Body weight change; Cardiac function: EF, FS; myocardial/inflammatory markers: NT-proBNP, BNP, cTnT, CK-MB, CRP, etc.; Histopathology: Myocardial morphology	No weight loss or cardiac/histopathological damage in rats; myocardial markers/inflammatory factors similar to controls, no obvious cardiotoxicity or inflammation at this dose	[132]
	H9C2 cardiomyocytes	In vitro	Baicalin 50 μM, treated for 24 h	Cell viability: Expression levels of signaling pathway proteins TLR4, IκBα, and p-p65/T-p65	No inhibition of cardiomyocyte viability; no activation of the TLR4/NF-κB pathway	
Allergic Asthma	Mice (OVA + LPS-induced allergic asthma)	Gavage	Baicalin 10, 25, and 65 mg/kg/day for 15 days (days 22–36 after sensitization)	Pulmonary organ coefficient; Histopathology; Body weight change and behavioral status	Dose-dependently reduced elevated lung coefficient, serum IgE, and BALF IL-17A/IL-6 induced by OVA + LPS; increased IL-10; alleviated lung/airway pathology; regulated Th17/Treg via STAT3/FOXP3; no toxicity in mice	[133]
Alcoholic Liver Disease	Zebrafish larvae (alcohol-induced alcoholic liver disease model)	Water exposure	Baicalin 6.25, 12.5, 25 μM for 48 h (after 32 h of 350 mM ethanol treatment)	Survival rate; Hatching rate; Heart rate; Body length; Morphological changes (pericardial edema, yolk residue, swim bladder absence, etc.)	No significant survival decrease (72–96 h post-fertilization [hpf]), normal hatching (48–72 hpf ~100%); heart rate/body length similar to normal controls; no malformations at ≤25 μM baicalin	[134]
	Human hepatocyte line LO2 (alcohol-induced injury model)	In vitro	Baicalin 6.25, 12.5, 25 μM, pretreated for 1 h followed by 100 mM ethanol treatment for 8 h	Cell viability; Lipid accumulation (Nile red staining); Fatty acid synthase (FASN) expression	No reduction in cell viability; improved liver health status as indicated by reduced lipid accumulation; 25 μM inhibited FASN without cytotoxicity	
	Mouse macrophage cell line RAW264.7	In vitro	Baicalin 6.25, 12.5, 25 μM, treated for 24 h	Cell morphology; Inflammatory factor secretion (IL-6, TNF-α, etc.)	Normal cell morphology, no apoptosis/necrosis; reduced IL-6/TNF-α, no abnormal proliferation from excessive immunosuppression	
Anti-bone Metastatic Breast Cancer	Human breast cancer cell lines (MDA-MB-231, MCF-7)	In vitro	Baicalin 1, 10, 100 nM, treated for 48 h	Cancer cell viability; Normal cell toxicity	Selectively inhibited breast cancer cell viability (100 nM most effective); no toxicity to human mesenchymal stem cells (hMSCs), slightly enhanced viability; no nonspecific death of other normal cells	[135]
	Mouse monocyte-macrophage cell line (RAW 264.7, induced into osteoclasts)	In vitro	Baicalin 1, 10, 100 nM, cotreated with RANKL for 5 days	Osteoclast survival rate; Number of tartrate-resistant acid phosphatase (TRAP)-positive cells	Dose-dependently reduced TRAP+ osteoclasts, toxic only to osteoclasts (low to undifferentiated RAW 264.7), no abnormal apoptosis/inflammation	
	Human bone marrow mesenchymal stem cells (hMSCs, induced into osteoblasts)	In vitro	Baicalin 1, 10, 100 nM, treated for 7 days	Osteoblast differentiation markers (ALP, OCN, and RUNX2 mRNA); Cell morphology	Low doses (1–10 nM) upregulated osteogenic genes and promoted differentiation; high dose (100 nM) slightly inhibited alkaline phosphatase (ALP), no abnormal morphology; no osteoblast necrosis/dysfunction	
	Mouse preosteoblasts (mMSCs)	In vitro	Baicalin 10, 100 nM, treated for 7 days	Osteogenic marker mRNA expression; Cell viability	No significant inhibition of osteoblast viability; 100 nM slightly downregulated OCN/ALP mRNA, no effect on survival; no morphology change or apoptosis	
Ketamine-induced Developmental Neurotoxicity	Neonatal rats (PND7, ketamine-induced neurotoxicity)	Intraperitoneal injection	Administered 30 min before ketamine injection (baicalin 25, 50, 100 mg/kg), once every 90 min, 5 times in total	Neuron morphology; Cell apoptosis; Caspase-3 activity and mRNA expression; PI3K/Akt pathway-related protein expression (p-Akt, p-GSK-3β)	100 mg/kg baicalin alleviated ketamine-induced neuronal damage, increased Nissl-positive neuron number; dose-dependently reduced TUNEL-positive cell number (100 mg/kg group decreased ~50%); inhibited caspase-3 activity/mRNA; activated PI3K/Akt, reversed p-Akt/p-GSK-3β downregulation, no excessive proliferation	[136]
	Primary rat cortical neuron-glia mixed culture (ketamine-induced injury)	In vitro	Baicalin 20, 50, 100 μM, pretreated for 30 min followed by 2 mM ketamine for 24 h	Cell viability; Cell morphology; Cleaved caspase-3 expression level	100 μM baicalin increased cell viability (20 μM had no effect); mitigated neuronal damage, increased nodes, slight glial damage; reduced cleaved caspase-3, effect blocked by PI3K inhibitor LY294002 (specific action)	
	Normal neonatal rats (PND7)	Intraperitoneal injection	Baicalin 100 mg/kg, administration schedule the same as the toxicity model group	Neuron morphology and apoptosis indicators; Basal activity of the PI3K/Akt pathway	No abnormal neuron morphology (Nissl/TUNEL same as normal) after single administration; no interference with PI3K/Akt, no excessive survival from overactivation	

## Data Availability

No new data were created or analyzed in this study. Data sharing is not applicable.

## Abbreviations

Ags, antigens; APCs, Antigen-presenting cells; BALF, bronchoalveolar lavage fluid; cagA, cytotoxin-associated gene A; cAMP, cyclic adenosine monophosphate; *C.difficile*, *Clostridioides difficile*; CDI, *C.difficile* infection; CFTR, cystic fibrosis transmembrane conductance regulator; cGMP, cyclic guanosine monophosphate; CLP, cecal ligation and puncture; CSF, cerebrospinal fluid; DCs, dendritic cells; DESs, Deep Eutectic Solvents; DMSO, Dimethyl Sulfoxide; EAE, Enzyme-Assisted Extraction; EDTA, ethylenediamine tetraacetic acid; eIF2, eukaryotic translation initiation factor 2; ETEC, Enterotoxigenic *Escherichia coli*; FZD7, frizzled class receptor 7; GUCA2A, guanylate cyclase activator 2A; GUCA2B, guanylate cyclase activator 2B; GUCY2C, guanylate cyclase C; HDT, Host-directed therapy; *H.Pylori*, *Helicobacter pylori*; HRAE, Heat-reflux-assisted extraction; IgA, immunoglobulin A; IgM, immunoglobulin M; IKKs, IκB kinases; IL-1, interleukin-1; IL-8, interleukin-8; LDH, lactate dehydrogenase; LPS, lipopolysaccharide; LT, labile enterotoxin; MAE, Microwave-Assisted Extraction; MDRSS, multidrug-resistant *Staphylococcus aureus*; MRP5, multidrug resistance-associated protein 5; MRSA, methicillin-resistant *Staphylococcus aureus*; *NHEs*, Na^+^/H^+^ exchangers; NIK, NF-κB-inducing kinase; NLRP3, nucleotide-binding oligomerization domain-like receptor family pyrin domain-containing 3; PERK, protein kinase R-like endoplasmic reticulum kinase; PKC, protein kinase C; PKG, protein kinase G; PMF, proton motive force; SEAP, secreted alkaline phosphatase; SIRS, systemic inflammatory response syndrome; ST, stable enterotoxin; TCA, tricarboxylic acid; TcdA, enterotoxin A; TcdB, cytotoxin B; TNF-α, tumor necrosis factor-α; TLR4, Toll-like receptor 4; TXNIP, thioredoxin-interacting protein; T4SS, Type IV Secretion System; UAE, Ultrasonic-Assisted Extraction; UHPE, Ultrahigh-Pressure Extraction; *vacA*, vacuolating cytotoxin gene A.
